# Internet Addiction in Chinese Adolescents in Hong Kong: Assessment, Profiles, and Psychosocial Correlates

**DOI:** 10.1100/tsw.2008.104

**Published:** 2008-08-07

**Authors:** Daniel T. L. Shek, Vera M. Y. Tang, C. Y. Lo

**Affiliations:** ^1^ Centre for Quality of Life, Hong Kong Institute of Asia-Pacific Studies, The Chinese University of Hong Kong, Hong Kong; ^2^ Kiang Wu Nursing College of Macau, Macau; ^3^ Social Welfare Practice and Research Centre, The Chinese University of Hong Kong, Hong Kong; ^4^ Jockey Club Wah Ming Lutheran Integrated Service Centre, Hong Kong Lutheran Social Service, LC-HKS, Hong Kong

**Keywords:** Internet addiction measurements, Chinese adolescents, Internet use and activities

## Abstract

Internet addiction behavior was examined in 6,121 Chinese primary and secondary school students in Hong Kong based on the assessment frameworks of Ivan Goldberg and Kimberly Young. Results showed that scales derived from both frameworks (CIA-Goldberg Scale and CIA-Young Scale) were internally consistent and evidence supporting their validity was found. Descriptive statistical analyses revealed that roughly one-fifth of the respondents could be classified as Internet addicted based on either scale. Further analyses showed that Internet-addicted and -nonaddicted respondents differed in their Internet use and related behavior. Logistic regression analyses showed that engagement in certain on-line activities (such as playing on-line games and downloading software) and replacement of pastimes activities (such as watching TV and going out with friends) with Internet activities predicted a higher probability of Internet addiction.

## INTRODUCTION

The Internet has become a basic tool for trading, entertainment, and communication, as well as education, in the contemporary world. Nevertheless, despite its positive uses, the presence of additive behaviors among some Internet users has drawn the attention of the public and helping professionals. In 1995, Ivan Goldberg borrowed criteria for defining psychoactive substance dependence in the DSM-IV and coined the term "Internet Addiction Disorder" to represent those having problematic Internet use behavior[1]. Since then, much research and debates about Internet addiction have been generated.

Similarly, as a leading expert in the field of problematic Internet use behavior, Kimberly Young developed the 7-, 8-, 10-, and 20-item scales for assessing "Internet dependent" or "Internet addicted" behavior[2,3,4,5]. In one of her studies with a sample of 596 Internet users[1], Young found that 66% of the respondents could be classified as "Internet dependent", with the addictive behaviors including tolerance, loss of control, withdrawal, and functional impairment, which caused negative academic, social, financial, and occupational consequences[1,2]. There are other attempts to construct Internet addiction measurements. For example, Brenner developed a 32-item questionnaire entitled "Internet-Related Addictive Behavior Inventory" (IRABI) to assess Internet addiction and found that most of the respondents experienced at least one of the Internet addiction symptoms, including tolerance, craving, and withdrawal[1]. Egger and Rauterberg[6] also designed a questionnaire to examine Internet behaviors and addiction tendency of 454 Internet users in Switzerland and other countries, and reported that 10.6% of the respondents were addicted to or dependent on the Internet.

Studies about Internet addiction have also been conducted in Chinese communities. In a study of 910 college students in Taiwan, Chou and Hsiao[7] used the 40-item Chinese Internet-Related Addictive Behavior Inventory version II (C-IRABI-II) and Young's 8-item questionnaire to examine Internet addiction among the respondents. They found that 5.9% of the respondents could be classified as addicted to the Internet. In another study of 2,203 adolescents from Taiwan, Ng et al.[8] adopted the 26-item Chinese Internet Addiction Scale and found that 10.7% of the respondents could be regarded as potentially at risk of Internet addiction, while 3.5% could be identified as seriously at risk of Internet addiction. In a study of 293 respondents from Fuzhou in mainland China, Lin and Yan[9] used the modified Young's 10-item questionnaire, which showed that 9.6% of the respondents could be regarded as addicted to the Internet.

In Hong Kong, utilizing the Young's 20-item questionnaire to examine Internet addiction among 976 students, the Tsuen Wan Centre of the Chinese YMCA of Hong Kong[10] found that 61.4% of senior primary school students, 35.2% of Secondary 1 to 3 students, 18.8% of Secondary 4 to 5 students, 35.8% of Secondary 6 to 7 students, and 37.0% of college students could be identified as highly at risk of Internet addiction. By comparing the data derived from two studies using the Young's 8-item questionnaire, Chan[11] found that the prevalence of Internet addiction had increased from 3.0% in 2000 to 14.7% in 2002. In another study of 1,182 adolescents, Yip and Kwok[12] used the modified Young's 8-item questionnaire, which showed that 5.4% of the respondents were on-line game addicts.

There are also research findings showing that adolescents experience the negative impact of Internet-addictive behaviors. For example, in a study of 1,716 Secondary 1 to 3 students, Against Child Abuse[13] found that 37% of the respondents admitted that they "could not resist the attraction from being on-line" and 28.5% of the respondents agreed that "on-line activities largely affected their normal daily lives". Similarly, in a study of 677 pairs of parent-child, Choi et al.[14] found that 14.5% of the parent respondents often had conflicts with their children regarding on-line problems.

Regarding school performance, it was found that Internet-addicted college students rated the impact of the Internet on their study to be more negative than did their nonaddicted counterparts[7]. Parents also thought that there was negative impact on the academic performance of their children if they spent over 4.15 h on the Internet daily[15]. Finally, regarding the impact on psychosocial well-being, Internet addiction was found to be associated with subjective distress and social impairment[16]. Pratarelli et al.[17] argued that increased Internet use was associated with feelings of isolation, which were mutually reinforcing. Choi et al.[14] also found that 17.1% of the adolescent respondents who spent more than 6 h on-line daily had difficulty in controlling their unstable emotions.

There are several limitations pertinent to the existing literature on Internet addiction problems among adolescents in Chinese societies. First, few validated instruments are available, and not many attempts have been made to develop and validate the assessment tools for Internet addiction. Obviously, without valid and reliable assessment tools, it is difficult for helping professionals to identify adolescents with Internet addiction problems and develop intervention models to help them. Second, because most of the available studies were based on small samples, our understanding of Internet addiction problems among Chinese adolescents remains limited. Third, only few studies have examined the relationship between Internet use behavior (e.g., playing on-line games) and Internet addiction.

Against the above background, Internet addiction among primary and secondary school students was examined in this study using assessment tools based on the frameworks of Ivan Goldberg and Kimberly Young in a Chinese context (i.e., CIA-Goldberg Scale and CIA-Young Scale). Several research questions are addressed in this paper:

1. What are the psychometric properties of the CIA-Goldberg Scale and CIA-Young Scale?
2. What are the profiles of responses to the items in the CIA-Goldberg Scale and CIA-Young Scale?
3. What are the differences between Internet-addicted and -nonaddicted adolescents in terms of Internet use and related behavior?
4. What factors predict Internet addiction in young people in Hong Kong?

The findings reported in this paper were based on a project entitled "Youngster Internet Addiction Prevention and Counseling Service" funded by the Community Chest of Hong Kong, which aimed to provide preventive and counseling services for young people who displayed Internet addiction problems.

## METHOD

### Participants

A convenient sample with 6,121 students recruited from 16 primary and secondary schools in Hong Kong was used. Most of the respondents were aged between 11 and 18 years (97.7%). The three dominant age groups in the sample included the 15–16 group (25.5%), 13–14 group (37.6%), and 11–12 group (21.8%). There were slightly more males (51.2%) than females (48.8%) in the sample. Regarding the duration that the participants had been using the Internet, 18.6% had used for 3.0–3.9 years, 17.2% had used for 4.0–4.9 years, 20% had used for 5.0–5.9 years, and less than 5% of the sample had been using the Internet for 8 years or above at the time of the study.

### Instruments

Each respondent was invited to complete a self-administered questionnaire that contained different measures of Internet addiction symptoms, Internet usage, and background information (e.g., family income, academic performance and conduct at school, perceived quality of family life). The questionnaire is available on request from the first author.

#### *Assessment of Internet Addiction*

To give a comprehensive assessment of Internet addiction, two measures were used in this study. These included:

1. The Chinese Internet Addiction (Goldberg) Scale (CIA-Goldberg) — In 1995, Ivan Goldberg borrowed the criteria of substance abuse in the DSM-IV and proposed seven criteria to assess whether a person can be defined as having "Internet Addiction Disorder"[18]. A person who meets three or more criteria is defined as having "Internet Addiction Disorder". The first and second authors translated the criteria into Chinese.
2. The Chinese Internet Addiction (Young) Scale (CIA-Young) — Kimberly Young developed several versions of assessment tools, including Young's 7-item questionnaire, Young's brief 8-item questionnaire, and Young's 10-item questionnaire. The items in the first and second assessment tools were developed with reference to the criteria for pathological gambling in the DSM-IV and psychoactive substance dependence in the DSM-IV, respectively[2,4]. Young's 10-item questionnaire[3] was employed in this study. A person who answers "Yes" to four or more of the questions is defined as "Internet dependent". The items were translated by the first and second authors.

### *Assessment of Use of the Internet*

Based on a review of the existing literature in both Western and Chinese communities[6,17,19], 13 items were developed by the first and second authors to understand the frequency of Internet use and activities engaged (e.g., playing on-line games, downloading software, meeting new friends) in the last 12 months among the respondents. In addition, two Internet addiction/dependence questions based on self-assessment were developed: "Do you consider yourself to be addicted to the Internet?" and "Do you consider that you excessively use or depend on the Internet to satisfy different kinds of needs?" The respondents gave their answers on a 5-point scale (1 = *definitely not* to 5 = *absolutely yes*).

### Procedures

The data for the present paper were collected in the context of school talks. After the third author of the present study and/or his colleagues obtained consent of the 16 schools, data were collected from students before the school talk on the topic related to healthy Internet usage, which was delivered by a social worker in each school. The purpose of the study was mentioned and the confidentiality of data collected was repeatedly emphasized to all of the students in attendance on the day of testing. The students participated in the study on a voluntary basis. They were asked to indicate their wish if they did not want to participate in the study (i.e., "passive"' informed consent was obtained from the students). At least one school staff member (most of them were teachers) was present throughout the test administration session. Adequate time was provided for the respondents to complete the questionnaire in self-report format. The respondents generally took 20–30 min to complete the questionnaire.

## RESULTS

### Psychometric Properties of CIA-Goldberg and CIA-Young

Results showed that the internal consistency of CIA-Goldberg and CIA-Young was good. Cronbach's alpha for CIA-Goldberg and CIA-Young was 0.68 and 0.75, respectively. Regarding validity of the scales, findings based on Pearson correlation analyses showed that both scales were moderately related to whether one was self-assessed as an Internet addict and whether there was excessive usage of or dependence on the Internet. The scale scores were also significantly related to the time spent on Internet activities (see Table 1).

**TABLE 1**
**Relationships between Two Assessment Tools of Internet Addiction (CIA-Goldberg and CIA-Young) and Other Measures of Excessive Internet Use***

| | CIA-Goldberg | CIA-Young |
|---|---|---|
| Self-assessed as "addicted to the Internet" | 0.406 | 0.423 |
| Self-assessed as "excessively use or depend on the Internet" | 0.393 | 0.412 |
| Average amount of total Internet time per week | 0.188 | 0.216 |
| Average amount of Internet time spent on entertainment per week | 0.186 | 0.207 |

* $p < 0.001$ (Bonferroni-corrected alpha level = 0.006).

## Profiles of Responses to CIA-Goldberg and CIA-Young

The percentages of responses to the questions in CIA-Goldberg and CIA-Young are presented in Tables 2 and 3, respectively. Using the cutoff score of 3, 22.9% ($N$ = 1,371) of the respondents could be classified as Internet addicted using the CIA-Goldberg. On the other hand, 19.1% ($N$ = 1,150) of the respondents could be identified as Internet addicted based on the CIA-Young.

**TABLE 2**
**Percentages of Responses to the Questions in CIA-Goldberg**

| Criterion | Question | *N* | % | |
|---|---|---|---|---|
| | | | Yes | No |
| 1 | (a) Do you find there is a diminished effect with continued use of the same time spent on the Internet? | 6,106 | 13.3 | 86.7 |
| | (b) Do you experience a tolerance, in that you have a need for increased amounts of Internet use to achieve the desired effect? | 6,121 | 21.9 | 78.1 |
| 2 | Do you access the Internet more often or for longer periods of time than you intended? | 6,102 | 32.2 | 67.8 |
| 3 | Have you had a persistent desire or made unsuccessful efforts to cut down or control the amount of time spent on-line? | 6,081 | 16.4 | 83.6 |
| 4 | Do you spend a great deal of time in activities related to Internet use (e.g., buying Internet books, trying out new WWW browsers, organizing files of downloaded materials)? | 6,102 | 26.6 | 73.4 |
| 5 | Have you given up or reduced any important social, occupational, academic, or recreational activities because of the Internet use? | 6,099 | 18.0 | 82.0 |
| 6 | Have you continued to use the Internet despite knowledge of having physical, social, occupational, academic, or psychological problems (e.g., sleep deprivation, interpersonal conflicts, lateness for work, lateness for school, neglect of student or occupational duties)? | 6,102 | 18.1 | 81.9 |
| 7 | (a) Have you made attempts to cut down on the excessive amount of time you spend on-line? | 5,942 | 42.4 | 57.6 |
| | (b) Have the following conditions been developed within several days to a month after you made attempts to cut down on the excessive amount of time you spend on-line? | | | |
| | I I have had psychomotor agitation. | 5,751 | 6.0 | 94.0 |
| | II I have felt anxious. | 5,751 | 3.0 | 97.0 |
| | III I have had obsessive thinking about what is happening on the Internet. | 5,751 | 9.9 | 90.1 |
| | IV I have had fantasies or dreams about the Internet. | 5,751 | 6.0 | 94.0 |
| | V My fingers have done typing movements voluntarily or involuntarily. | 5,751 | 2.1 | 97.9 |
| | (c) Have you used the Internet as a way of relieving or avoiding the above unwell conditions? | 5,745 | 4.8 | 95.2 |
| | (d) Do you feel that the above conditions cause distress or impairment in your social, occupational, or another important area of functioning? | 5,718 | 4.3 | 95.7 |
| **Overall "Internet Addicted" Percentage** (met three or more criteria) | | 6,000 | **22.9** | 77.1 |

**TABLE 3**
**Percentages of Responses to the Questions in CIA-Young**

| | Question | *N* | % Yes | % No |
|---|---|---|---|---|
| 1 | Do you feel preoccupied with the Internet or on-line services and think about it while off-line? | 6,118 | 25.2 | 74.8 |
| 2 | Do you feel a need to spend more and more time on-line to achieve satisfaction? | 6,121 | 21.9 | 78.1 |
| 3 | Have you repeatedly made unsuccessful efforts to control, cut back, or stop Internet use? | 6,104 | 17.6 | 82.4 |
| 4 | Do you feel restless, moody, depressed, or irritable when attempting to cut down or stop Internet use? | 6,106 | 12.3 | 87.7 |
| 5 | Do you stay on-line longer than originally intended? | 6,118 | 41.4 | 58.6 |
| 6 | Have you jeopardized or risked the loss of a significant relationship, job, educational or career opportunity because of the Internet? | 6,114 | 22.5 | 77.5 |
| 7 | Have you lied to family members, teachers, social workers, or others to conceal the extent of involvement with the Internet? | 6,102 | 11.3 | 88.7 |
| 8 | Do you use the Internet as a way of escaping from problems or of relieving a dysphoric mood (e.g., feelings of helplessness, guilt, anxiety, depression)? | 6,105 | 12.0 | 88.0 |
| 9 | Do you keep returning even after spending too much money on on-line fees? | 6,103 | 15.9 | 84.1 |
| 10 | Do you feel depressed, irritable, moody, or anxious when you are off-line? | 6,114 | 11.2 | 88.8 |
| **Overall "Internet Addicted" Percentage** (answered "Yes" to four or more questions) | | 6,024 | **19.1** | 80.9 |

## Differences between Internet-Addicted and -Nonaddicted Respondents

Results showed that the Internet-addicted and -nonaddicted groups could be differentiated in terms of the related scale scores (Table 4). Using CIA-Goldberg, the addicted group displayed a higher score than did the nonaddicted group (mean = 3.896 and 0.689 for the Addicted Group and Nonaddicted Group, respectively; t = 101.754, $p < 0.001$; Cohen's $d = 0.95$). Using CIA-Young, the addicted group displayed a higher score than did the nonaddicted group (mean = 5.511 and 1.061 for the Addicted Group and Nonaddicted Group, respectively; t = 86.997, $p < 0.001$; Cohen's $d = 1.39$). Results further showed several phenomena:

1. Internet-addicted respondents spent more time on-line than their nonaddicted counterparts.
2. Internet-addicted respondents made more new acquaintances solely on the Internet than their nonaddicted counterparts.
3. Internet-addicted respondents belonged to more clubs or organizations on the Internet than their nonaddicted counterparts.
4. Internet-addicted respondents engaged in various kinds of Internet activity more frequently than their nonaddicted counterparts.
5. Internet-addicted respondents had a stronger tendency to replace different kinds of off-line activities or pastimes with Internet activities than their nonaddicted counterparts.

**TABLE 4**
**Comparison of Internet Usage between Internet-Addicted (IA) and Internet-Nonaddicted (IN) Respondents**

| | | Mean | | | |
|---|---|---|---|---|---|
| | | Tool 1 (C-Goldberg) | | Tool 2 (C-Young) | |
| | | IA | IN | IA | IN |
| 1 | Average amount of Internet time spent on entertainment per week (in hours) | 15.8 | 9.1*** | 17.1 | 9.1*** |
| 2 | Average amount of Internet time spent on study per week (in hours) | 3.1 | 2.7 | 3.3 | 2.7* |
| 3 | Average amount of total Internet time per week (in hours) | 17.7 | 10.5*** | 19.5 | 10.5*** |
| 4 | Number of new acquaintances made solely on the Internet | 14.4 | 8.3 | 16.7 | 8.5* |
| 5 | Number of clubs or organizations you belong to on the Internet | 6.8 | 2.8** | 7.1 | 2.9** |
| 6 | Frequency of engaging in the following Internet activities (answered 1 = *never* to 4 = *very often*) | | | | |
| | I Playing on-line games | 3.18 | 2.70*** | 3.20 | 2.72*** |
| | II Downloading software | 2.97 | 2.49*** | 2.94 | 2.52*** |
| | III Talking to friends who immigrate to or study overseas (e.g., chat room, ICQ) | 2.75 | 2.43*** | 2.75 | 2.45*** |
| | IV Keeping track of new developments in the areas of personal interest (e.g., computer accessories, football, beauty, on-line games) | 3.05 | 2.69*** | 3.01 | 2.71*** |
| | V Downloading information (e.g., songs, movies, mobile phone ring tones) | 3.16 | 2.68*** | 3.13 | 2.71*** |
| | VI Reading and posting messages on newsgroup/discussion groups | 2.49 | 2.15*** | 2.48 | 2.16*** |
| | VII Meeting new friends | 2.37 | 1.92*** | 2.45 | 1.92*** |
| | VIII Updating personal homepage | 2.05 | 1.78*** | 2.07 | 1.80*** |
| | IX Seeking advice on different areas of life (e.g., interpersonal relationship, health) from professionals | 1.94 | 1.76*** | 1.97 | 1.76*** |
| | X Talking to on-line friends who share my interests | 3.05 | 2.52*** | 3.07 | 2.54*** |
| | XI WWW-surfing, browsing | 3.34 | 3.11*** | 3.29 | 3.13*** |
| 7 | Level of Internet replacing the following activities or pastimes (answered 1 = *never* to 4 = *very often*) | | | | |
| | I Watching TV | 2.98 | 2.73*** | 2.99 | 2.74*** |
| | II Talking to friends in person | 2.49 | 2.28*** | 2.50 | 2.29*** |
| | III Going out with family | 2.32 | 2.17*** | 2.37 | 2.16*** |
| | IV Participating in activities inside or outside school | 2.21 | 2.06*** | 2.20 | 2.07*** |
| | V Going out with friends | 2.48 | 2.20*** | 2.50 | 2.20*** |

* $p < 0.05$.

** $p < 0.005$.

*** $p < 0.001$.

## Predictors of Internet Addiction

Logistic regression analyses consistently showed that several predictors increased the likelihood of being classified as Internet addicted (Tables 5 and 6). These factors included certain on-line activities (such as playing on-line games, downloading software, and meeting new friends) and replacement of leisure time activities with Internet activities.

**TABLE 5**
**Logistic Regression of Activities Engaged On-Line and Activities or Pastimes Replaced with Internet, Based on Tool 1 (CIA-Goldberg)**

| Variables in the Equation | B | S.E. | Wald | df | Sig. | Exp(B) |
|---|---|---|---|---|---|---|
| Internet-related activities | | | | | | |
| Searching information for school-related assignments | –0.282 | 0.048 | 34.176 | 1 | 0.000 | 0.754 |
| Reading and writing e-mails | –0.119 | 0.042 | 7.875 | 1 | 0.005 | 0.888 |
| Playing on-line games | 0.345 | 0.040 | 74.068 | 1 | 0.000 | 1.412 |
| Downloading software | 0.254 | 0.045 | 31.830 | 1 | 0.000 | 1.289 |
| Talking to friends overseas | –0.022 | 0.037 | 0.353 | 1 | 0.552 | 0.979 |
| Keeping track of new developments in the areas of personal interest | 0.147 | 0.042 | 12.572 | 1 | 0.000 | 1.159 |
| Downloading information | 0.142 | 0.041 | 12.226 | 1 | 0.000 | 1.153 |
| Reading and posting messages on newsgroup/ discussion groups | 0.064 | 0.038 | 2.757 | 1 | 0.097 | 1.066 |
| Meeting new friends | 0.149 | 0.041 | 13.409 | 1 | 0.000 | 1.161 |
| Updating personal homepage | 0.087 | 0.037 | 5.553 | 1 | 0.018 | 1.091 |
| Seeking advice from professionals | 0.029 | 0.046 | 0.397 | 1 | 0.529 | 1.029 |
| Talking to on-line friends | 0.164 | 0.039 | 17.441 | 1 | 0.000 | 1.178 |
| WWW-surfing, browsing | 0.059 | 0.046 | 1.619 | 1 | 0.203 | 1.061 |
| Replacement of leisure time activities with Internet activities | | | | | | |
| Watching TV | 0.192 | 0.037 | 26.345 | 1 | 0.000 | 1.212 |
| Talking to friends in person | 0.056 | 0.040 | 1.937 | 1 | 0.164 | 1.058 |
| Going out with family | 0.021 | 0.041 | 0.273 | 1 | 0.601 | 1.022 |
| Participating in activities inside or outside school | –0.015 | 0.037 | 0.163 | 1 | 0.686 | 0.985 |
| Going out with friends | 0.141 | 0.037 | 14.456 | 1 | 0.000 | 1.151 |

## DISCUSSION

Research findings varied a lot on the rates of Internet addiction among youth, probably because different questionnaires were used. Despite the development of these different questionnaires for assessing Internet addiction, items measuring the occurrence of tolerance, withdrawal, and impairment of psychosocial functioning were often included. Some researchers argued that it was not appropriate to define Internet addiction as a unique psychiatric disorder, as the validity of the construct was doubtful. For instance, the existence of a wide range of Internet activities suggested that Internet addiction was unlikely to be a single phenomenon. In addition, the question about comorbidity conditions suggested that Internet addiction might just be an expression of other disorders[20]. Grohol[21] argued that people would go from overuse to balance eventually, while the overuse was only a transitional phase. These arguments suggest that reliable and valid assessment tools that can capture the dynamic nature of Internet addiction are badly needed.

**TABLE 6**
**Logistic Regression of Activities Engaged On-Line and Activities or Pastimes Replaced with Internet, Based on Tool 2 (CIA-Young)**

| Variables in the Equation | B | S.E. | Wald | df | Sig. | Exp(B) |
|---|---|---|---|---|---|---|
| Internet-related activities | | | | | | |
| Searching information for school-related assignments | –0.380 | 0.051 | 54.760 | 1 | 0.000 | 0.684 |
| Reading and writing e-mails | –0.096 | 0.045 | 4.554 | 1 | 0.033 | 0.908 |
| Playing on-line games | 0.355 | 0.043 | 67.498 | 1 | 0.000 | 1.426 |
| Downloading software | 0.193 | 0.048 | 16.244 | 1 | 0.000 | 1.213 |
| Talking to friends overseas | –0.020 | 0.039 | 0.274 | 1 | 0.601 | 0.980 |
| Keeping track of new developments in the areas of personal interest | 0.123 | 0.044 | 7.679 | 1 | 0.006 | 1.131 |
| Downloading information | 0.079 | 0.043 | 3.291 | 1 | 0.070 | 1.082 |
| Reading and posting messages on newsgroup/ discussion groups | 0.060 | 0.041 | 2.176 | 1 | 0.140 | 1.062 |
| Meeting new friends | 0.259 | 0.043 | 36.930 | 1 | 0.000 | 1.296 |
| Updating personal homepage | 0.094 | 0.039 | 5.800 | 1 | 0.016 | 1.099 |
| Seeking advice from professionals | 0.080 | 0.048 | 2.730 | 1 | 0.099 | 1.083 |
| Talking to on-line friends | 0.162 | 0.043 | 14.443 | 1 | 0.000 | 1.176 |
| WWW-surfing, browsing | –0.019 | 0.049 | 0.148 | 1 | 0.700 | 0.981 |
| Replacement of leisure time activities with Internet activities | | | | | | |
| Watching TV | 0.171 | 0.040 | 18.306 | 1 | 0.000 | 1.187 |
| Talking to friends in person | 0.034 | 0.043 | 0.633 | 1 | 0.426 | 1.035 |
| Going out with family | 0.108 | 0.043 | 6.263 | 1 | 0.012 | 1.114 |
| Participating in activities inside or outside school | –0.075 | 0.040 | 3.589 | 1 | 0.058 | 0.927 |
| Going out with friends | 0.171 | 0.039 | 18.732 | 1 | 0.000 | 1.186 |

In addition, many researchers on Internet addiction collected their data through Internet surveys, such as via e-mails and the World Wide Web (WWW), which led to the problems of sampling bias. For instance, in a review of Internet-based survey research, Brenner[22] pointed out that one significant bias of Internet addiction surveys was the over-representation of male respondents. In addition, the small sample size of the available studies was another issue: 76 respondents in Li and Chung's study[23], 100 respondents in Nalwa and Anand's study[24], and 271 respondents in Wang's study[25]. All these observations call for the development of reliable and valid Internet addiction measures through careful research designs.

In this study, the psychometric properties of two developed measures of Internet addiction were examined through 6,121 students, with similar proportions of male and female respondents. Results showed that the CIA-Goldberg and CIA-Young were internally consistent. In addition, the evidence for the construct validity of the two developed measures of Internet addiction were found, including the positive relationships between the CIA-Goldberg and CIA-Young, and measures of (1) self-assessment of Internet addiction, (2) self-assessment of excessive use of or dependence on the Internet, (3) amount of time spent on the Internet, and (4) amount of Internet time spent on entertainment. With reference to the concern of the lack of reliable and valid assessment tools for Internet addiction, the present study is a significant addition to the Chinese literature on Internet addiction among young people.

Based on the CIA-Goldberg and CIA-Young, 22.9 and 19.1% of the respondents in this study could be identified as Internet addicted, respectively. These results were similar to the results found in some of the existing studies targeting adolescents[26,27]. As Internet addiction adversely affects one's physical health, family life, and academic performance[28], and can cause severe psychological distress[29], the present findings alert us to the need for early identification of adolescents with Internet addiction and those at high risk for Internet addiction.

Several differences were found between the respondents in the Internet-addicted and -nonaddicted groups identified by CIA-Goldberg and CIA-Young in this study. First, the Internet-addicted respondents spent more time on-line than the nonaddicted respondents, which is consistent with the findings of previous studies[30,31]. Second, the Internet-addicted respondents made more new acquaintances on the Internet, a finding that is consistent with the results shown in Egger and Rauterberg's study[6]. Third, the Internet-addicted respondents belonged to more clubs or organizations on the Internet than the nonaddicted respondents, a finding that echoes the results shown in the study of Whang et al.[32]. Fourth, consistent with the previous findings[7,33], the Internet-addicted respondents engaged in various kinds of Internet activity more frequently than the nonaddicted respondents.

Based on the two measurements of Internet addiction in this study, it was found that the frequency of engaging in on-line activities, including playing on-line games, downloading software, meeting new friends, and talking to on-line friends, was a powerful predictor of Internet addiction. In addition, the frequency of leisure-time activities replaced with Internet activities, including watching TV and going out with friends, was also a powerful predictor of Internet addiction. In other words, the more frequently one engages in the above-mentioned on-line activities, and replaces watching TV and going out with friends with Internet activities, the more likely he or she will be addicted to the Internet. As there are few research studies examining the predictors of Internet addiction in Chinese adolescents, the present findings can be regarded as pioneering additions to the literature.

It is noteworthy that there are several limitations of the present study. First, as a convenient sample was used, generalizability of the present findings to the population should be addressed with caution. Second, the findings should be interpreted with reference to the debates surrounding the validity of the construct of Internet addiction. Third, if resources permit, Internet addiction based on ratings by psychiatrists should be added to further assess the criterion-related validity of the developed tools. Finally, as the significant correlation among the variables may be due to common method variance, further work utilizing a multitrait/multimethod strategy should be carried out. Despite these limitations, the present findings provide some pioneering findings on Internet addiction among Chinese adolescents in Hong Kong.

## ACKNOWLEDGMENTS

The Youngster Internet Addiction Prevention and Counseling Service undertaken by the Jockey Club Wah Ming Lutheran Integrated Service Centre, Hong Kong Lutheran Social Service, LC-HKS was funded by the Community Chest of Hong Kong. Preparation for this paper was financially supported by the Wofoo Foundation Limited.
